# Mutations in the *gdpP* gene are a clinically relevant mechanism for β-lactam resistance in meticillin-resistant *Staphylococcus aureus* lacking *mec* determinants

**DOI:** 10.1099/mgen.0.000623

**Published:** 2021-09-06

**Authors:** Anna Sommer, Stephan Fuchs, Franziska Layer, Christoph Schaudinn, Robert E. Weber, Hugues Richard, Mareike B. Erdmann, Michael Laue, Christopher F. Schuster, Guido Werner, Birgit Strommenger

**Affiliations:** ^1^​ Department of Infectious Diseases, Nosocomial Pathogens and Antibiotic Resistances, Robert Koch Institute, Wernigerode, Germany; ^2^​ Methodology and Research Infrastructure, Bioinformatics, Robert Koch Institute, Berlin, Germany; ^3^​ Centre for Biological Threats and Special Pathogens, Advanced Light and Electron Microscopy, Robert Koch Institute, Berlin, Germany

**Keywords:** c-di-AMP, *gdpP *mutations, GWAS, MRSA, penicillin-binding proteins

## Abstract

In *Staphylococcus aureus *, resistance to β-lactamase stable β-lactam antibiotics is mediated by the penicillinbinding protein 2a, encoded by *mecA* or by its homologues *mecB* or *mecC*. However, a substantial number of meticillin-resistant isolates lack known *mec* genes and, thus, are called meticillin resistant lacking *mec* (MRLM). This study aims to identify the genetic mechanisms underlying the MRLM phenotype. A total of 141 MRLM isolates and 142 meticillin-susceptible controls were included in this study. Oxacillin and cefoxitin minimum inhibitory concentrations were determined by broth microdilution and the presence of *mec* genes was excluded by PCR. Comparative genomics and a genome-wide association study (GWAS) approach were applied to identify genetic polymorphisms associated with the MRLM phenotype. The potential impact of such mutations on the expression of PBP4, as well as on cell morphology and biofilm formation, was investigated. GWAS revealed that mutations in *gdpP* were significantly associated with the MRLM phenotype. GdpP is a phosphodiesterase enzyme involved in the degradation of the second messenger cyclic-di-AMP in *S. aureus *. A total of 131 MRLM isolates carried truncations, insertions or deletions as well as amino acid substitutions, mainly located in the functional DHH-domain of GdpP. We experimentally verified the contribution of these *gdpP* mutations to the MRLM phenotype by heterologous complementation experiments. The mutations in *gdpP* had no effect on transcription levels of *pbp4*; however, cell sizes of MRLM strains were reduced. The impact on biofilm formation was highly strain dependent. We report mutations in *gdpP* as a clinically relevant mechanism for β-lactam resistance in MRLM isolates. This observation is of particular clinical relevance, since MRLM are easily misclassified as MSSA (meticillin-susceptible *S. aureus *), which may lead to unnoticed spread of β-lactam-resistant isolates and subsequent treatment failure.

## Data Summary

The sequence data generated during the study are available in the European Nucleotide Archive (https://www.ebi.ac.uk/ena), under study accession number PRJEB41705.

**Impact Statement**

The study described here is dedicated to the phenomenon of *mec*-independent β-lactam resistance, which has recently gained increased attention in the literature. It contributes additional insights and discussion points at the interface of microbial diagnostics, microbial genomics, antibiotic therapy and infection control. Using comparative genomics in combination with a genome-wide association study (GWAS) approach, we identified mutations in the c-di-AMP phosphodiesterase-encoding *gdpP* as causative for the resistance phenotype in a unique, comprehensive strain collection comprising exclusively patient isolates.

## Introduction

β-Lactam antibiotics, in particular isoxazolyl penicillins and first- and second-generation cephalosporins, are still the first choice for the treatment of staphylococcal infections. Their effect is based on allosteric binding of the β-lactam ring to native penicillin-binding proteins (PBPs). This results in the formation of an acyl-enzyme complex that prevents cross-linking of the peptidoglycan in the bacterial cell wall. This disrupts cell wall integrity, ultimately resulting in cell death. Meticillin-resistant *Staphylococcus aureus* (MRSA) usually carries an additional PBP, PBP2a, encoded by *mecA* or rarely by its homologues *mecB* or *mecC*. It is characterized by reduced affinity to most β-lactams; thus, allowing cross-linking in the presence of these antibiotics [1–3]. However, **m**eticillin-**r**esistant **l**acking ***m*** *ec* (MRLM) isolates have been described, but only limited data is available on their prevalence, characteristics and underlying resistance mechanisms [4–8]. Although these isolates seem to be rare at present, they nevertheless pose a serious threat to public health and therapeutics as they are easily misclassified as meticillin-susceptible *S. aureus* (MSSA) based on the exclusive detection of *mec* genes or PBP2a [9–14].

The MRLM phenotype has been previously associated with (i) a hyperproduction of *bla*Z-encoded β-lactamase (BORSA phenotype) [15], (ii) reduced affinity of native PBPs to β-lactams due to *pbp* mutations (MODSA phenotype) [16–19] or (iii) increased peptidoglycan cross-linking due to mutations in the *pbp4* promoter resulting in overexpression of PBP4 [20]. Additionally, it has been shown that loss-of-function mutations in *gdpP*, a gene involved in the metabolism of the second messenger cyclic-di-AMP, can lead to reduced susceptibility of *S. aureus* to β-lactams [4, 21, 22]. However, it remains unclear how common *gdpP* mutations are in clinical isolates, as previous reports include only limited numbers of clinical MRLM isolates (*n*_max_=20) [4, 5, 7, 23], or such with only slightly elevated minimum inhibitory concentration (MIC) values [23].

The use of genome-wide association studies (GWASs) to identify molecular mechanisms of antibiotic resistance has been successfully employed previously [24, 25]. In combination with comparative genomics, we used this approach to link phenotypes and genotypes of 141 clinical MRLM isolates collected across Germany between 2006 and 2019 and a control set of isolates, in order to identify lineage-independent polymorphisms associated with the MRLM phenotype.

## Methods

### Bacterial isolates

All isolates (*n*=283) originated from submissions to the German National Reference Centre (NRC) for Staphylococci between 2006 and 2019. They were subjected to antibiotic-susceptibility testing, PCR for the detection of *mecA* or *mecC*, and *spa*-typing [26, 27]. A total of 141 isolates phenotypically resistant towards oxacillin (OXA) (MIC >2 mg l^−1^) and cefoxitin (CXI) (MIC >4 mg l^−1^) but lacking a *mec* gene were selected and classified as ‘meticillin resistant lacking *mec*’ (MRLM, according to work published elsewhere [5]). In addition, 142 MSSA isolates were chosen as controls. Besides their resistance phenotype, isolates were selected to represent the current German *S. aureus* population with respect to clonal lineage and geographical origin (Fig. S1, available with the online version of this article) [28–31].

### Susceptibility testing

Susceptibility testing was performed using broth microdilution according to European Committee on Antimicrobial Susceptibility Testing (EUCAST) criteria (clinical breakpoints v.9.0) [32] for the following compounds: benzylpenicillin (BEN), oxacillin (OXA), cefoxitin (CXI), gentamicin (GEN), erythromycin (ERY), clindamycin (CLI), tetracycline (TET), vancomycin (VAN), teicoplanin (TEI), ciprofloxacin (CIP), trimethoprim/sulfamethoxazole (TRS), fusidic acid (FUS), rifampicin (RIF), mupirocin (MUP), fosfomycin (FOS), linezolid (LIN), moxifloxacin (MOX), tigecycline (TIG) and daptomycin (DAP). β-Lactamase hyperproduction (BORSA phenotype) was excluded by oxacillin/sulbactam (OXA-SU) screening [33].

### Whole-genome sequencing (WGS) and sequence assembly

Genomic DNA was extracted from overnight cultures in TSB (tryptic soy broth) using the DNeasy blood and tissue kit (Qiagen) and quantified with the Qubit dsDNA HS assay kit (Thermo Fisher Scientific) according to the manufacturers' instructions. Sequencing libraries were generated with the Nextera XT DNA library preparation kit (Illumina) and paired-end sequencing was performed using either a MiSeq or a HiSeq instrument with the 2×300 MiSeq v3 and the 2×250 HiSeq Rapid SBS v2 reagent kit (Illumina), respectively. The quality of raw reads was checked using FastQC v01.11.5 [34] and putative contaminations were assessed with Kraken v1.0 [35]. FQStat(S. Fuchs, personal communication) revealed a mean coverage of 226±116 fold. Trimmed raw reads (Trimmomatic v0.36) [36] were *de novo* assembled using SPAdes v3.12.0 [37] and the quality of assemblies was verified with quast v5.0 [38].

### WGS-based molecular typing and phylogenetic analyses

SeqSphere^+^ v7.1.0 (Ridom) was used to extract *spa*-type [39] (for confirmation of routine results), multilocus sequence type (MLST) [40] and core-genome MLST (cgMLST) complex type (CT) [41] from *de novo* assembled contigs. Based on cgMLST data, 49 192 SNPs in 1074 loci present in all isolates were used to calculate a neighbour-joining tree using Geneious Prime 2020.0.5 (Biomatters) that was further visualized with iTOL v4 [42]. The tree was annotated according to clonal complexes (CCs) using the stringent CC definition (six identical loci define a CC) [43]. For comparison reasons, phylogeny was further reconstructed based on whole-genome SNPs. Trimmed paired-end reads were aligned to the reference genome of *S. aureus* COL (NC_002951.2) using the in-house pipeline batchMap v2.0.0, as previously described [44]. The resulting consensus alignment was reduced to variant positions using SNPfilter v3.2.3 with default parameters and using an exclusion distance of 150 bp [45]. Based on the resulting SNP alignment, a neighbour joining tree was calculated in Geneious Prime 2020.0.5 (Biomatters).

### SNP and indel analysis

*De novo* assembled contigs of all sequenced isolates were aligned to the MSSA reference strain NCTC 8325 (NC_007795) using Geneious Prime 2020.0.5 (Biomatters). Amino acid substitutions/indels in the four native *pbp* genes (*pbp1–pbp4*), the *pbp4* promoter and the *gdpP* gene of each strain were compared, and non-silent polymorphisms were classified into lineage-specific and putatively MRLM-associated polymorphisms. All corresponding polymorphisms are listed in Fig. S1. Further sequence comparison of closely related MRLM–MSSA pairs (*n*=2) was carried out by aligning trimmed paired-end reads of the MSSA isolate to the concatenated, *de novo* assembled contigs of the corresponding MRLM isolate using the in-house pipeline batchMap as described above.

### GWAS and gene enrichment

The microbial pan-GWAS tool scoary v1.6.16 was used to link patterns of gene presence or absence with the MRLM phenotype [46]. For this, a presence/absence table of 2567 core and accessory genes based on *de novo* assembled contigs of all 283 isolates was generated using Seqsphere^+^ v7.1.0 (Ridom). A gene was defined as present if the corresponding allele was at least 95 % identical to the reference gene from *S. aureus* COL (NC_002951.2) and neither contained any frameshift nor truncation. Additionally, a binary trait matrix input was created containing the isolate’s resistance phenotype (MRLM vs MSSA). scoary was run with default parameters. The presence or absence of a gene was classified as associated with the MRLM phenotype if (i) the Benjamini–Hochberg adjusted *P* value was <0.05, (ii) the worst pairwise comparison *P* value was <0.05, and (iii) the empirical *P* value (based on 100 permutations) was <0.05. Finally, a Manhattan plot illustrating the significance levels of resistance-associated genes in relation to their position in the *S. aureus* COL reference genome was created. For illustration purposes, SNPs with a -log_10_ *P* value of 0 were excluded. In addition, a gene enrichment analysis was used to test whether some genes possessed significantly more mutations in MRLM isolates compared to MSSA isolates (with respect to the reference genome). For each gene, a negative binomial count model was created based on isolates possessing the MSSA phenotype. This model was used as a null model to test for enrichment of mutations in the MRLM isolates. The resulting *P* values were then adjusted using a Benjamini–Hochberg correction.

### Transcription levels of *pbp4*

TSB overnight cultures were diluted 1 : 100 into TSB medium and grown at 37 °C to an OD_600_ of 0.6. Aliquots of 20 ml were centrifuged for 10 min at 7000 ***g*** and 4 °C. Pellets were resuspended in 800 µl 1× RNA protection reagent (NEB) and cells were lysed mechanically for 3 min using 0.1 mm glass beads on a MM400 mixer mill (Retsch). Cell debris and glass beads were separated from the lysate by centrifugation at 12 000 ***g*** for 5 min, and RNA was extracted using the Monarch total RNA miniprep kit (NEB) following the manufacturer’s protocol. RNA integrity was verified using the Agilent 2100 Bioanalyzer with the 6000 Nano Kit (Agilent). Real-time PCR analyses were performed with the Luna universal one-step RT-qPCR kit (NEB) according to the manufacturer’s instructions and using the CFX connect real-time PCR detection system (Bio-Rad). Transcription levels of *pbp4* were determined as the mean of three biological replicates for each strain, utilizing the primers pbp4_forward (5´-CTGCATACGAACCGACGAGT-3´) and pbp4_reverse (5´-GTCATAGACGCTGGATTCCACT-3´). Δ*C*q values were determined by normalization to the transcription levels of the 16S rRNA gene for each strain utilizing the primers 16S_forward (5´-TCAACCTTGCGGTCGTACTC-3´) and 16S_reverse (5´-CACGCCGTAAACGATGAGTG-3´). Finally, the *pbp4* transcription level of the reference strain NCTC 8325 (NC_007795) was defined as baseline and fold-change values for each strain were calculated. Unpaired *t*-tests were done to determine significant differences in transcription levels of *pbp4* using GraphPad Prism (v8.4.0).

### Biofilm formation

TSB overnight cultures were diluted 1 : 100 into fresh TSB medium supplemented with 1 % (w/v) glucose. Cell suspension (200 µl) was aliquoted into sterile 96-well flat-bottom plates and incubated at 37 °C for 24 h. Adherent bacteria were washed three times with 1x PBS, dried for 1 h at room temperature and stained with 100 µl of a 0.1 % (w/v) crystal violet solution for 5 min. The wells were washed three times with 1x PBS and adhering cells were resuspended with 100 µl of 5 % (v/v) acetic acid and the *A*_620_ was measured. Values for each strain were calculated relative to the MRSA reference strain 38887 (10–03022) [47]. Biofilm levels were determined in three biological replicates with three technical replicates each. Unpaired *t*-tests were done to determine significant differences in biofilm formation using GraphPad Prism (v8.4.0).

### Analysis by electron microscopy

TSB overnight cultures were diluted 1 : 100 into fresh TSB medium and grown at 37 °C to an OD_600_ of 0.6. Aliquots (20 ml) were centrifuged at 5000 ***g*** for 5 min. The pellet was resuspended in 0.05 M HEPES buffer (pH 7.5) and centrifuged at 5000 ***g*** for 2 min. Finally, the pellet was dissolved in 1 ml clinical fixative (2.5 % glutaraldehyde, 1 % paraformaldehyde, 0.05 M HEPES buffer pH 7.5) and incubated for at least 48 h.

For transmission electron microscopy (TEM) imaging, staphylococcal cells were embedded in agarose by mixing equal volumes of cell suspension and low-melting-point agarose (3 % in distilled water). After post-fixation (1 % OsO4 in distilled water, 1 h), staining (2 % uranyl acetate in distilled water, 1 h), stepwise dehydration in a graded ethanol series and embedding in LR White resin (Science Services), the blocks were polymerized at 60 °C overnight. Ultra-thin sections were prepared using a ultramicrotome (UCT; Leica Microsystems) and then counterstained with uranyl acetate (2 % in distilled water) for 10 min and lead citrate for 3 min. All sections were examined in a transmission electron microscope (Tecnai12, FEI) operated at 120 kV.

For scanning electron microscopy (SEM) imaging, circular 12 mm coverslips were coated with 1 % Alcian Blue to facilitate bacterial adhesion, loaded with 100 µl *S. aureus* cell suspension and incubated for 30 min at room temperature in a moisture chamber. Subsequently, the coverslips were washed, dehydrated in a graded ethanol series, critical point dried, mounted on aluminium stubs, sputter coated with an 8 nm layer of gold/palladium and examined with a scanning electron microscope (ZEISS 1530 Gemini; Carl Zeiss Microscopy) operating at 3 kV with the in-lens electron detector at 10 000-fold magnification.

The diameters of 100 individual cells per strain were measured at their widest circumference using Fiji [48]. Only those bacteria were measured that presented themselves in a not tilted, unambiguous angle to the viewer. Unpaired *t*-tests were done to determine significant differences in cell sizes using GraphPad Prism (v8.4.0).

### *gdpP* complementation

For complementation of *gdpP,* five clinical MRLM isolates from CCs CC1 and CC30 carrying different mutations in *gdp*P were selected (Table 1, Fig. S1). We used the anhydrotetracycline (ATc)-inducible expression vector pRAB11 [49], in which the *bla* gene was deleted to prevent hyperproduction of β-lactamase leading to OXA resistance (pRAB11∆*bla*). The *gdpP* gene was amplified from NCTC 8325 genomic DNA with primers *gdpP*-pRAB11-for (5´-TTGATAGAGTATGATGGTACCACTGACACCTACGACACATAT TG-3´) and *gdpP*-pRAB11-rev (5'-TTGTAAAACGACGGCCAGTGAATTCTACCTTTACCT TTACCTTTAACATC-3'). The resulting PCR product was cloned into pRAB11∆*bla* that had been digested with *Eco*RI and *Kpn*I by Gibson assembly according to the manufacturer’s protocol (NEB). The resulting plasmid pRAB11∆*bla-gdpP* was passed through *Escherichia coli* IM01B or IM30B [50] depending on the CC of the final recipient, and then used to electroporate the clinical *S. aureus* MRLM isolates. Expression of *gdpP* was induced with 100 ng ATc ml^−1^. OXA MICs were determined by broth microdilution and were compared to the corresponding isolates containing the empty plasmid pRAB11∆*bla*.

**Table 1.** OXA MICs of MRLM strains complemented with a functional *gdpP* gene

Isolate	CC	*gdpP*	MIC OXA (mg l^−1^)			
			WT	+ATc (100 ng ml^−1^)		
				WT†	pRAB11 ∆*bla*	pRAB11 ∆*bla-gdpP*
17–01004	1	DEL77	8	4	4	1
19–00273	1	Q101R; Q224*	4	4	4	1
15–03617	1	INS1042	8	8	8	0.25
10–02437	30	DEL1099	4	4	4	0.5
17-01691-2	30	A9D; DEL342	4	4	4	0.5

†The MIC was determined with the addition of 100 ng ATc ml^−1^.

*, Stop codon.

## Results and Discussion

### Characterization of the strain collection

#### Clinical origin and phenotypic susceptibility

**Fig. 1.**
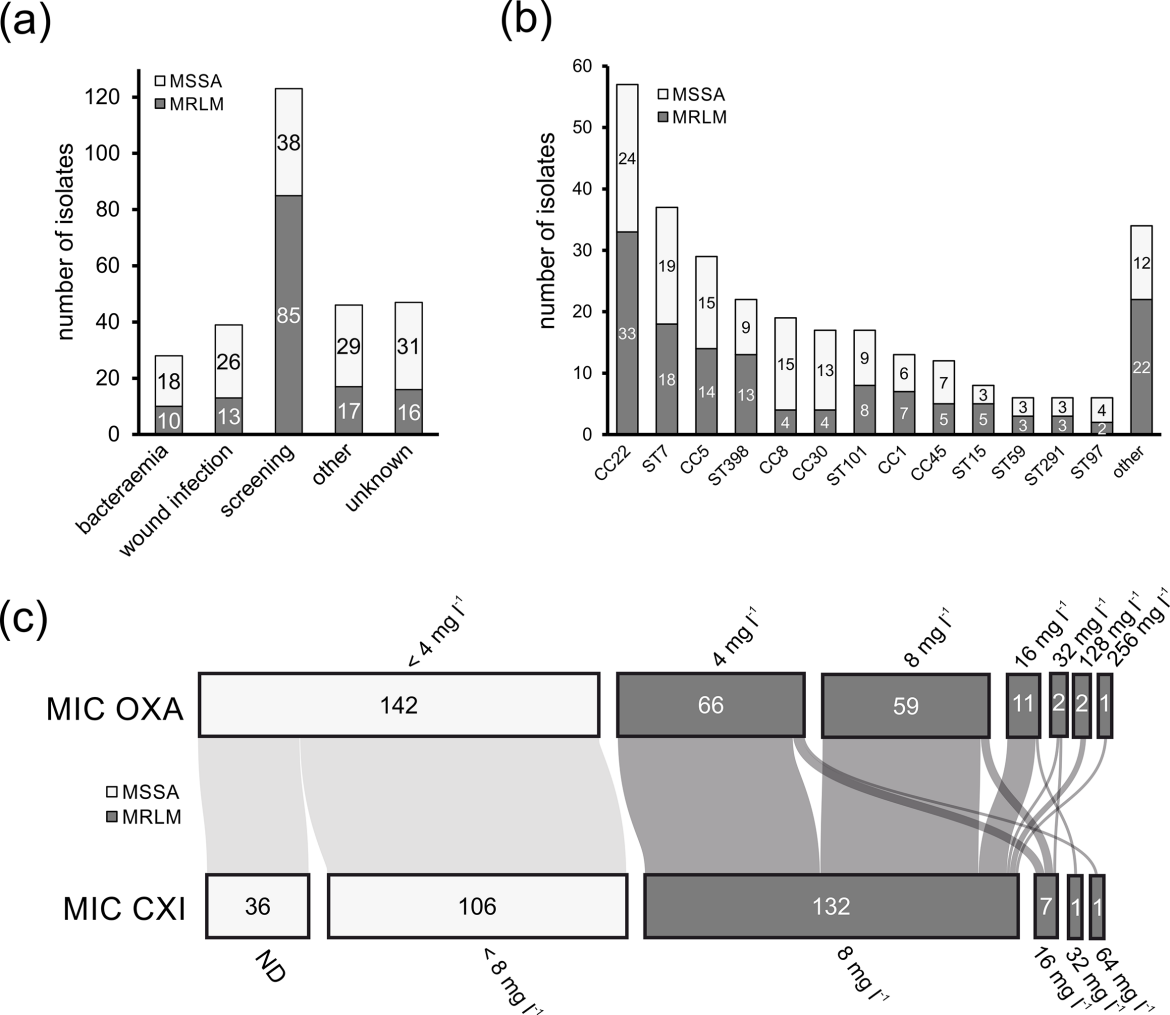
(**a**) Clinical origin, (**b**) CCs and MLSTs, and (**c**) phenotypic susceptibility of *S. aureus* MRLM (*n*=141; dark grey) and MSSA (*n*=142; light grey) isolates. ND, not determined. Flow between categories is indicated by connecting lines. Line width is proportional to quantity.

A total of 141 MRLM and 142 MSSA were obtained from human (*n*=280) and animal (*n*=3) samples, originating from various clinical diseases including bacteraemia (*n*=28), wound infections (*n*=39), pneumonia (*n*=6), urinary tract infections (*n*=6) and abscesses (*n*=12). A substantial number of isolates originated from screenings (*n*=123) and from samples with unknown clinical origin (*n*=47) (Fig. 1a). The MRLM phenotype did not appear to be associated with any clinical appearance, as MRLM and MSSA strains were encountered across clinical diseases with similar frequencies (Fig. 1a, b). Therefore, we do not have evidence that MRLM isolates occur more frequently in severe chronic infections and might be selected as a consequence of prolonged β-lactam therapy, as has been suggested recently [23]. Based on our results, it appears that the selection of MRLM occurs in the community and is potentially due to a hitherto unknown selection pressure (e.g. the use of antiseptics or disinfectants as recently proposed [51]). However, we have not systematically collected the isolates investigated in this study and are not in possession of the full meta-data, so this conclusion remains speculative. Further research on the conditions of the MRLM selection process is, therefore, necessary.

Most MRLM isolates showed narrow resistance patterns (Fig. S2) and borderline resistant MICs for OXA (MIC 4–8 mg l^−1^; *n*=125) and CXI (MIC 8 mg l^−1^; *n*=132). Only a few isolates exhibited OXA MICs from 16 to 256 mg l^−1^ (*n*=16) and CXI MICs from 16 to 64 mg l^−1^ (*n*=9) (Fig. 1c). All MRLM isolates were resistant to OXA in the presence of oxacillin/sulbactam (OXA-SU), suggesting that β-lactamase overproduction is not involved in MRLM phenotype expression. Furthermore, within our MRLM collection, a large proportion of isolates were *blaZ* negative. Therefore, a link between MRLM phenotype development and *blaZ* presence could not be confirmed as suggested in another report (Fig. S2) [23]. Also, based on the genome sequences, the presence of *mecB* could be excluded as a cause for the MRLM phenotype [1].

#### Phylogeny

**Fig. 2.**
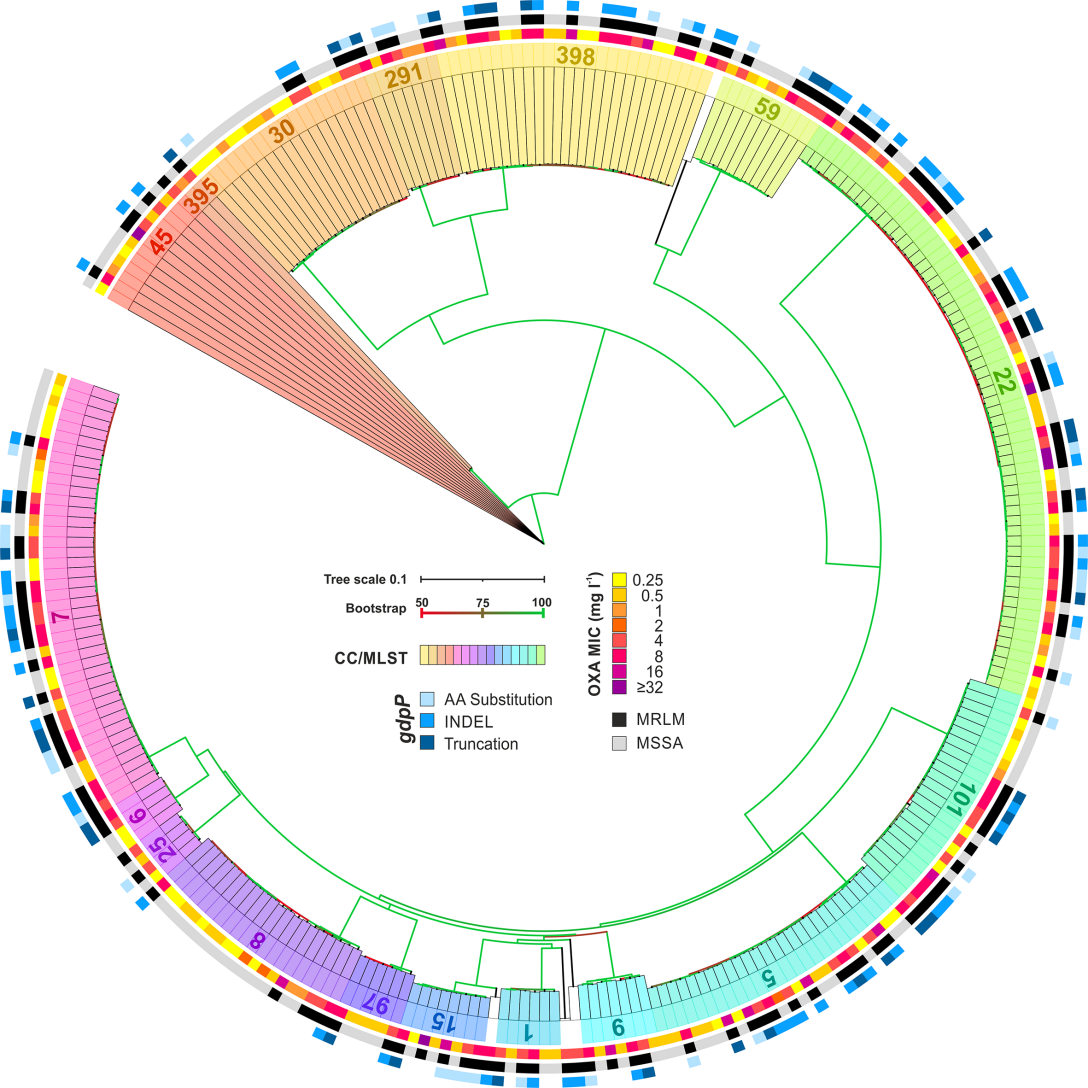
Phylogenetic analysis of *S. aureus* MRLM (*n*=141) and control isolates (*n*=142). The neighbour-joining tree is based on 49 192 SNPs in 1074 cgMLST loci present in all isolates. It is colour coded by CC and MLST. The inner circle indicates the OXA MIC. MRLM isolates are indicated by black boxes and control isolates by light grey boxes in the middle circle. The outer circle indicates isolates with amino acid (AA) substitutions (light blue), INDELs (insertion or deletion) (blue) or truncations (dark blue) in the *gdpP* gene. The scale bar indicates the proportion of sequence differences. Branch colour indicates bootstrap support.

Extraction of *spa*-types (*n*=73) and MLSTs (*n*=46) from WGS data confirmed that the strain collection included a wide range of clonal lineages (Figs 1b and 2, Fig. S1).

MRLM and MSSA strains were evenly distributed throughout a neighbour-joining tree based on 49 192 SNPs in 1074 cgMLST loci (Fig. 2). This result could be confirmed by the use of SNP-based neighbour joining trees within individual CCs (data not shown). Based on these results, we concluded that the strain collection is suitable for the application of GWAS. It has previously been shown that the reliability of GWAS is highly dependent on the quality of the strain collection. The use of a well-balanced strain collection minimizes the risk of detecting polymorphisms associated with clonal lineage rather than with the phenotype of interest. Moreover, including a wide range of clonal lineages enables the detection of convergent mutations in different clonal lineages as a consequence of selection [52, 53].

cgMLST analysis revealed two MRLM–MSSA pairs within the strain collection. Isolates of each pair were obtained from a single patient at one time point, indicating the presence of a heterogenous population within the patients. The isolates showed no genetic differences within the 1074 cgMLST loci employed for phylogenetic reconstruction. Isolates of the first pair belonged to sequence type (ST)59 [12–03487 (MRLM) and 12–03488 (MSSA)] and were isolated from an abscess of a human host, while the other two isolates were assigned to ST1 [12–00973 (MRLM) and 12–00975 (MSSA)] and originated from a horse suffering from an eczema. Subsequent read mapping revealed that both MRLM isolates harboured non-synonymous mutations in *gdpP* when compared to the corresponding MSSA isolate. MRLM isolate 12–03487 additionally harboured a T616A substitution in the *fnbA* gene.

#### Mutations in *gdpP*

**Fig. 3.**
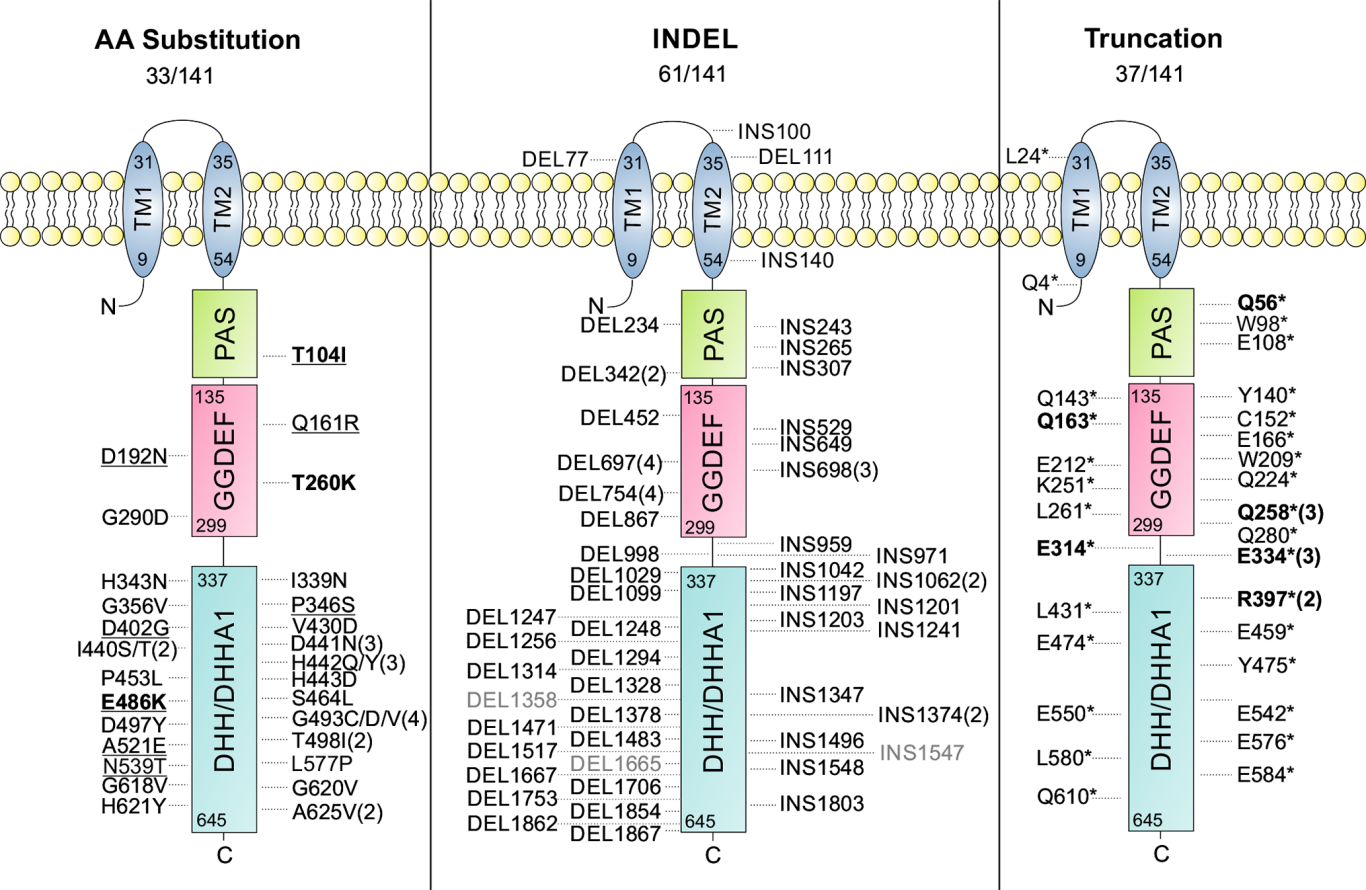
Schematic representation of the GdpP protein in 141 *S. aureus* MRLM isolates. Locations of amino acid (AA) substitutions and truncations are given according to their amino acid position, whereas INDELs are given as nucleotide positions. Amino acid substitutions categorized as tolerated based on sift analysis are underlined, INDELs not leading to frameshifts are in grey and mutations previously described as associated with the MRLM phenotype are in bold. *, Stop codon; DEL, deletion; INS, insertion. Domain annotations are based on InterPro (https://www.ebi.ac.uk/interpro/protein/UniProt/Q2G2T6/) [71].

*gdpP* encodes the **G**GDEF-**d**omain **p**rotein **p**hosphodiesterase, GdpP, consisting of two N-terminal trans-membrane domains followed by three intracellular domains [54]. The N-terminal DHH-domain is required for the degradation of the second messenger cyclic-di-AMP into phosphadenylyl-adenosine (pApA) [22]. In the MRLM isolate 12–00973 of pair one, GdpP was truncated at position R397*, while the isolate 12–03487 of pair two revealed a H442Q mutation in the active-site DHH-domain of GdpP (Fig. 3).

Comparative analysis of all available datasets revealed mutations in *gdpP* in 131 of the MRLM isolates of our collection (92.9 %). This included truncations (*n*=37), insertions or deletions (*n*=61), mostly leading to frameshifts (*n*=58), as well as diverse amino acid substitutions (*n*=33) mainly located in the functional DHH-domain of the protein (Fig. 3, Fig. S1). Further analysis of respective amino acid substitutions using sift (Sorting Intolerant from Tolerant) [55] revealed that at least 24 (72.7 %) of the 33 amino acid substitutions were predicted to affect protein function (Fig. 3). In contrast, most susceptible control isolates carried a GdpP variant that matched the GdpP of the meticillin-sensitive *S. aureus* strain NCTC 8325 (Fig. S1). This indicated that loss-of-function mutations in the *gdpP* gene might be causal for the meticillin-resistance phenotype in the majority of MRLM isolates investigated. Mutations in *gdpP* causing reduced susceptibility to β-lactams have been previously reported in *S. aureus* laboratory mutants [21–23], as well as in smaller clinical strain collections [4, 5, 7]. In detail, the following polymorphisms found in MRLM isolates in our strain collection have been previously associated with β-lactam resistance: T104I [16], T260K [21], E468K, Q56*, Q163*, Q258*, E314* [5], E334* and R397* [4]. The amino acid substitutions D105N [5, 16], P392S [5], I456V and D561E [56] have also been associated with β-lactam resistance previously, but were found in our collection amongst MRLM as well as control isolates, suggesting that they are rather associated with clonal lineage than with the MRLM phenotype (Fig. S1).

In addition to β-lactam resistance in *S. aureus *, *gdp*P mutations have also been implicated in DAP non-susceptibility in enterococci. However, in a previous study applying GWAS to a collection of DAP-resistant *S. aureus *, no association to mutational changes in *gdp*P was observed [57]. Furthermore, in the strain collection described here, none of the MRLM isolates examined were DAP resistant.

#### Mutations in *pbp1–pbp4* and the *pbp4* promoter

β-Lactam resistance in MRLM strains has been previously associated with various mutations in the native *pbp1–pbp4* genes [4, 5, 7, 17–20]. In our strain collection, 44 (31.2 %) of 141 isolates carried a total of 57 different amino acid substitutions that occurred exclusively in MRLM strains [PBP1 (*n*=14); PBP2 (*n*=19); PBP3 (*n*=16); PBP4 (*n*=8)]. Among these isolates, 40 (91 %) carried mutations in *gdpP* as well. Seven of these *pbp* mutations were detected previously and have been associated with the MRLM phenotype (H499Y [5, 7] and P100T [4] in PBP1; T284I [5] and S569A [4, 19] in PBP2; P233L [16] and S634F [5, 7] in PBP3; and R200L [4, 5, 20] in PBP4).

Additionally, 34 amino acid substitutions previously described to be associated with β-lactam resistance were found in MRLM isolates and in controls, again suggesting that they are associated with a clonal lineage rather than the MRLM phenotype (Fig. S1). This finding highlights the need for case and control isolates from a wide range of epidemiologically relevant clonal lineages to exclude lineage-specific polymorphisms.

Six MRLM isolates contained neither mutations in the native *pbp1–pbp4* genes nor in *gdpP*. Among these, a single isolate carried a G-to-A mutation 9 bp upstream of the start codon of *gdpP*, which could reduce the expression of GdpP and eventually lead to phenotypic β-lactam resistance, in a manner similar to loss-of-function mutations in GdpP. In two other isolates, deletions in the *pbp4* promoter were identified. One of these isolates exhibited a 17.4-fold increased transcription of *pbp4* (Table 2). Overexpression of PBP4 has previously been described to cause β-lactam resistance by increasing the amount of cross-linked peptidoglycan [20, 58]. Increased amounts of cross-linked peptidoglycan, as well as increased transcription levels of the native *pbp4*, were also reported in a *gdpP* laboratory mutant [59]. However, in this study, comparative analysis of *pbp4* transcription levels revealed no differences between MRLM isolates with mutations in *gdpP* and their corresponding control isolates (Table 2). Similarly, no differences in expression levels of PBP4 were found in a *dacA* (encoding c-di-AMP cyclase) mutant strain expressing decreased levels of c-di-AMP [60]. These results indicate that in clinical *gdpP* mutants increased transcription of *pbp4* is not involved in the MRLM phenotype. Thus, the link between increased c-di-AMP levels and the MRLM phenotype remains unclear and putative mechanisms involving c-di-AMP-dependent regulation of cell wall homeostasis and cell wall stress response need to be further investigated.

**Table 2.** *pbp4* transcription levels of *S. aureus* MRLM and MSSA isolates in comparison to the MSSA reference strain NCTC 8325

Isolate	MLST	MIC OXA (mg l^−1^)	MIC CXI (mg l^−1^)	*gdpP*	*pbp4* promoter	*pbp4* transcription (FC)	*P* value
14–01665	ST45	32	16	–	11 bp DEL at −298 bp	14.97 (±3.23)	0.0036
12–00973	ST1	4	8	R397*	–	1.01 (±0.32)	ns
12–00975		0.5	<4	–	–	0.89 (±0.31)	
12–03487	ST59	4	8	H442Q	–	1.67 (±0.51)	ns
12–03488		0.5	<4	–	–	0.86 (±0.19)	

FC, Fold-change; ns, not significant.

*, Stop codon

### GWAS

**Fig. 4.**
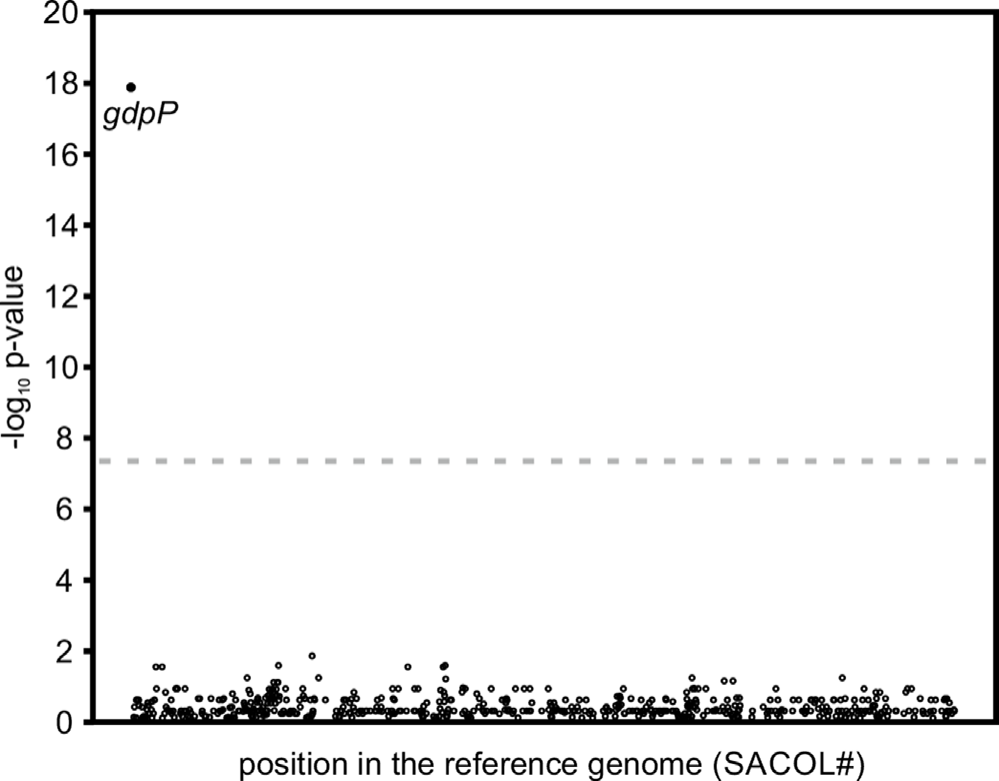
MRLM-associated loci determined by scoary GWAS. Worst pairwise comparison -log_10_ *P* values of all analysed loci are plotted against their corresponding location in the *S. aureus* COL reference genome (SACOL#). For the purpose of illustration, loci with a -log_10_ *P* value of zero were excluded. The dashed line indicates the genome-wide significance threshold (*P* value=5×10^−8^).

To further evaluate the significance of mutations in *gdpP* as the possible main resistance mechanism in clinical MRLM isolates, we applied a GWAS approach. GWAS revealed the absence of a functional GdpP as significantly associated with the MRLM phenotype (naïve *P*=1.7×10^−37^; Benjamini–Hochberg corrected *P*=2.8×10^−34^; worst-pairwise comparison *P*=8.2×10^−19^; empirical *P*=0.0099; Fig. 4).

In 140 of 142 control isolates, *gdpP* was identified as being present (Fig. S1). In contrast, in 96 of 141 MRLM isolates *gdpP* was absent, which is consistent with results from the detailed *gdpP* sequence analysis. Manual sequence analysis of the remaining 45 MRLM revealed 32 different amino acid substitutions in GdpP in 33 isolates (Fig. 3). These amino acid substitutions did not result in *gdpP* being assessed as ‘not present’ in these isolates, although amino acid substitutions might have an influence on the functionality of GdpP and, therefore, on the resistance phenotype.

To identify putative resistance determinants aside from *gdpP*, we initially also applied the reference-free, *k*-mer based dbGWAS algorithm [61] and the SNP-based GWAS-tool plink [62]. None of these approaches revealed *k*-mers or SNPs significantly associated with the MRLM phenotype (data not shown). In the case of the *gdpP* and *pbp* genes, this is most likely due to the large diversity of polymorphisms across the collection of MRLM isolates.

The high diversity of mutations in the *gdp*P gene is likely to complicate the establishment of rapid genotypic tests for the detection of *gdp*P-mediated β-lactam resistance. Ultimately, only sequencing of the gene or the entire genome can provide reliable information on the MRLM status based on comparison to MSSA isolates. Furthermore, based on our data, a small proportion of the phenotype may also be due to other only partially known polymorphisms, which makes easy and rapid molecular diagnostics even more difficult.

**Fig. 5.**
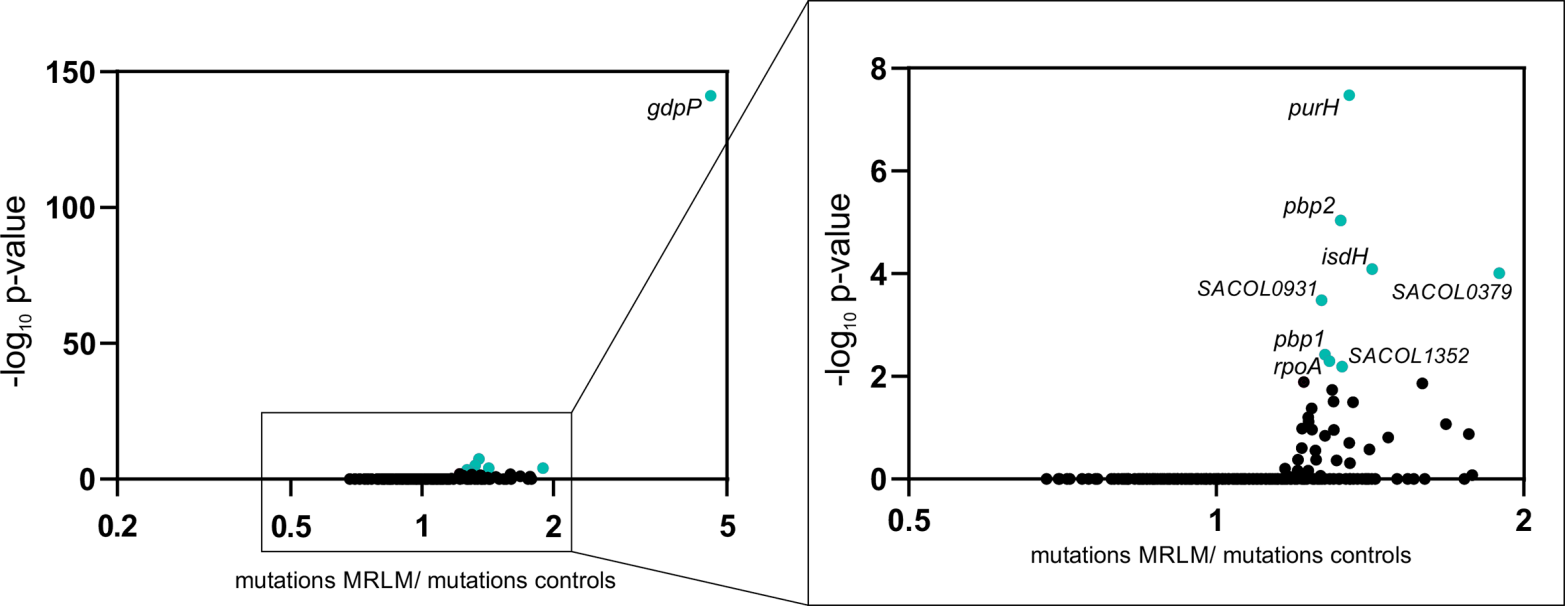
MRLM-associated loci determined by gene-enrichment analysis. Benjamini–Hochberg corrected -log_10_ *P* values of all analysed genes were plotted against the ratio of mutations in MRLM versus control isolates. Genes with a significantly enriched number of mutations in MRLM isolates are highlighted in turquoise (*P* value <0.01).

We conducted a gene-enrichment analysis to determine whether some genes possessed significantly more mutations in MRLM isolates compared to control isolates (Fig. 5). The *gdpP* gene had, on average, 4.6 times more mutations in MRLM strains compared to MSSA controls. In addition, eight other genes were significantly enriched with *P* values <0.01, but the increase in mutation ratios were below 1.5-fold. These genes also included *pbp*1 and *pbp*2, which were previously associated with β-lactam-resistance [4, 5, 7, 19].

Our GWAS results highlight the importance of determining the appropriate GWAS tool, as identification might be highly dependent on the type of genetic differences (e.g. horizontal acquisition of genes, mobile genetic elements vs mutations vs indels, truncations) and their distribution [25]. It is important to keep in mind that GWAS generally does not reveal causality but statistical associations with the phenotype of interest. However, in this study, GWAS results support the findings of comparative genomics and mutation enrichment studies. In contrast to our results, Giulieri *et al*. [23] used various approaches to find a variety of different genes associated with the MRLM phenotype. However, due to differences in strain collections and analyses, the results from the comparative analysis of clinical isolates, selection experiments and GWAS were only partially congruent, but also pointed to *gdpP* being associated with the MRLM phenotype [23].

### Genetic complementation

To experimentally test whether the proposed mutations in *gdpP* indeed mediate the observed β-lactam resistance, we cloned a functional *gpdP* gene from *S. aureus* 8325 into an inducible expression plasmid. The complementation plasmid was transferred to five MRLM strains containing different *gdpP* mutations (from two distinct CCs) and resistance to β-lactam antibiotics was tested as before.

MIC values of the WT strains with added ATc were slightly lower than without the addition, presumably due to low basal toxicity of the inducer. Induced heterologous expression of the functional *gdpP* gene alongside the native, mutant variants in the MRLM strains led to a complete restoration of the β-lactam-sensitive phenotype (Table 1). While the induced wild-type MRLM isolates containing the empty plasmid displayed MIC values from 4 to 8 mg l^−1^, MICs of the complemented isolates ranged from 0.25 to 1 mg l^−1^. This corresponds to a reduction of 3 to 5 dilution steps. Thus, in addition to showing association by GWAS, we could also experimentally demonstrate that the identified *gdp*P mutations are responsible for the MRLM phenotype in these strains.

### Biofilm formation

The biofilm phenotype of clinical isolates strongly contributes to the virulence and resistance properties of *S. aureus* [63]. For several bacterial species, it has been demonstrated that an intracellular accumulation of c-di-AMP promotes biofilm formation [64]. However, reported results for *gdpP* mutants in *S. aureus* were contradictory [22, 65, 66]. Within our strain collection we analysed possible effects of *gdpP* mutations on biofilm formation in 11 MRLM and 11 MSSA isolates.

**Fig. 6.**
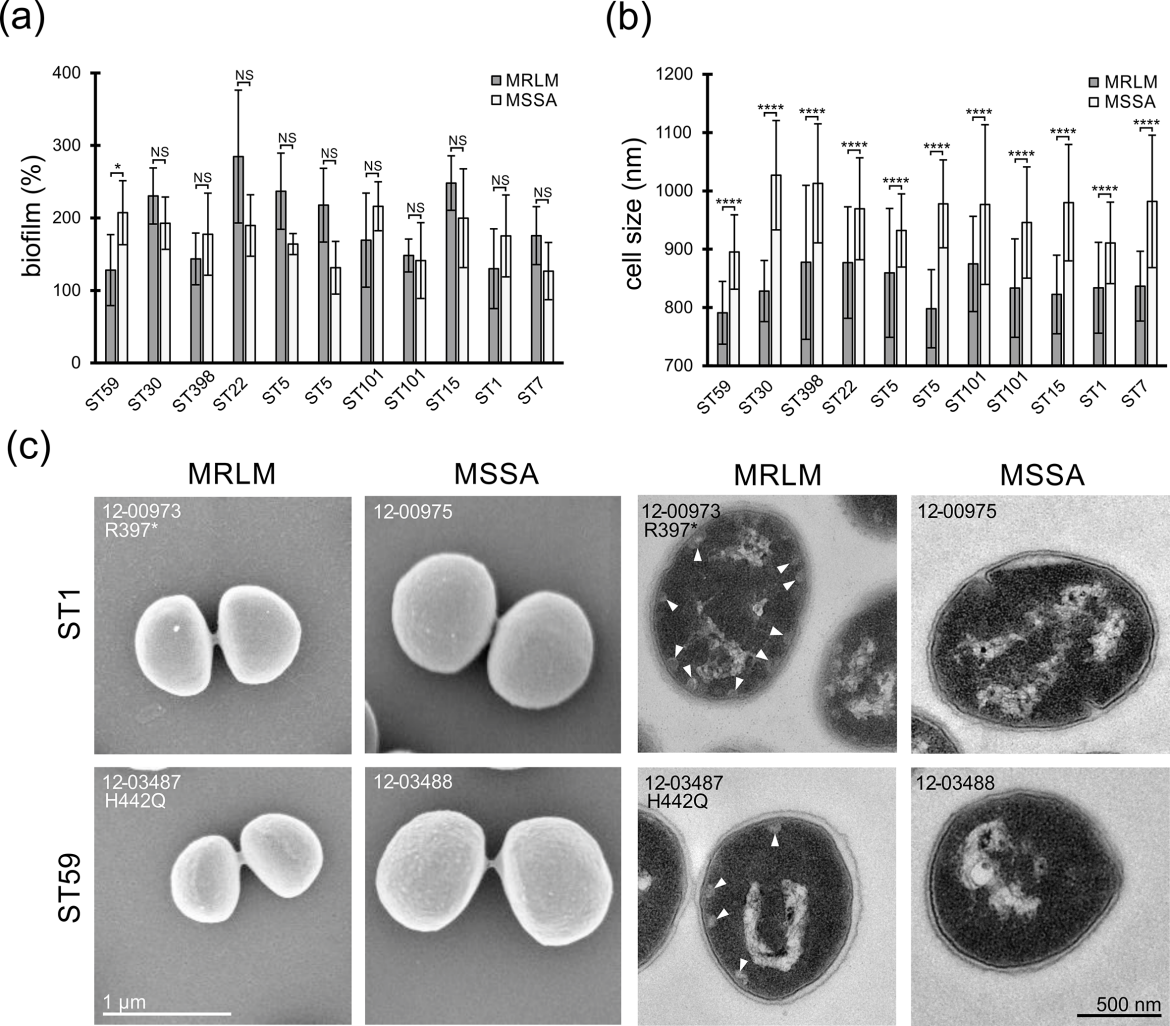
(**a**) Biofilm formation capacity of *S. aureus* MRLM and MSSA isolates relative to the MRSA reference strain 38887 (10–03022) [47]. (**b**) Mean cell sizes of *S. aureus* MRLM and MSSA isolates. (**c**) Electron microscopy of *S. aureus* MRLM isolates from two different lineages compared to corresponding MSSA controls. Size differences demonstrated in the SEM figures (left) and membrane associated vacuole-like structures indicated by white triangles in the TEM figures (right). NS, not significant. *, *P*<0.05; ****, *P*<0.0001.

The subset of strains covered different clonal lineages, as well as diverse mutations in *gdpP* (Table 3). We found no consistent link between the MRLM phenotype and biofilm-forming abilities (Fig. 6a, Table 3), which is in line with previous studies on biofilm formation in *gdpP* mutants. While Corrigan *et al.* [22] reported inconsistent effects of *gdpP* mutations on the ability to form biofilms depending on the genetic background, Chung *et al.* [66] demonstrated an increase in biofilm formation with a mutation in *gdpP*. In contrast, DeFranscesco *et al.* [65] reported a reduction of biofilm formation along with a reduced eDNA (extracellular DNA) release in *gdpP*-mutated strains. In summary, these results suggest that the influence of c-di-AMP levels on biofilm formation is likely dependent on the strain background.

**Table 3.** Phenotypic and genotypic characteristics of *S. aureus* MRLM and MSSA isolates

Isolate	MLST	MIC OXA (mg l^−1^)	MIC CXI (mg l^−1^)	*gdpP*	Cell size (nm)	Biofilm (%)
12–03487†	ST59	4	8	H442Q	791	128
12–03488†		0.5	<4	–	895	207
12–00973†	ST1	4	8	R397*	834	130
12–00975†		0.5	<4	–	911	175
16–03119	ST398	8	8	Q4*	877	143
16–02227		0.25	<8	–	1013	178
16–01876	ST22	4	8	DEL111	877	285
10–00598		0.25	nd	–	969	190
16–01897	ST5	4	8	INS649	859	237
14–02523		0.25	<8	–	932	164
19–00165	ST5	16	8	L580*	798	218
15–01414	ST4411	0.25	<8	–	978	131
13–03200	ST106	4	16	Y475*	875	169
16–00130	ST101	0.5	<8	–	977	216
13–03730	ST101	8	8	DEL1256	833	148
07–01215		0.5	nd	–	946	141
19–00062	ST15	16	8	D441N	822	248
08–01750		0.5	nd	–	980	200
17–01791	ST7	8	8	Y140*	837	127
16–02745		0.5	<8	–	982	176

DEL, Deletion; INS, insertion; nd, not determined; *, stop codon.

†Pairs including closely related isolates from a single patient.

### Cell size and morphology

Analysis of the same subset of 22 strains via SEM revealed clinical MRLM isolates with mutations in *gdpP* (*n*=11) to be reduced in cell size in comparison to MSSA isolates (*n*=11) (MRLM mean diameter=839±29 nm, MSSA mean diameter=964±38 nm; Fig. 6b, Table 3). MRLM isolates were further analysed by thin-section TEM to reveal additional morphological effects of *gdpP* mutations. In contrast to MSSA isolates, MRLM isolates contained membrane-associated vesicles in the cytoplasm (Fig. 6c). The reduced cell size and presence of membrane-associated vesicles in a *gdpP* deletion mutant were also described by Corrigan *et al.* [22]. This supports the hypothesis that mutations in *gdpP* could be causal for those morphological differences. However, the role of membrane-associated vesicles is still unexplained.

The influence of intracellular c-di-AMP levels on cell shape has been investigated previously. Increased c-di-AMP levels have been shown to inhibit the uptake of osmolytes, finally leading to a reduction of cell turgor [22, 67, 68]. Cell turgor reduction has been shown to cause decreased growth rates and cell sizes in Gram-positive bacteria, which might explain the smaller cell sizes reported [69, 70]. Additionally, the cell turgor reduction of *gdpP* mutants might be associated with the MRLM resistance phenotype, since it prevents cell burst mediated by cell-wall-targeting antibiotics like β-lactams [70]. A decrease in growth rates, suggesting a relevant fitness cost of the MRLM phenotype, was seen in many isolates of our collection and also was reported by Giulieri *et al.* [23].

### Conclusions

In summary, we report mutations in *gdpP* as an important mechanism for low-level β-lactam resistance in clinical MRLM isolates. Based on statistical analyses, we could demonstrate a significant association between *gdpP* mutations and resistance phenotype, and could experimentally verify this for a selection of strains. However, there is currently no knowledge of how this particular β-lactam resistance genotype has been selected. The current guidelines for MRSA diagnostics require confirmation of an MRSA phenotype by detecting either a *mec* gene or the corresponding protein PBP2a. This is not possible for MRLM isolates, which leads to inconclusive results in clinical MRSA diagnostics. However, a correct identification of β-lactam resistances is essential in order to provide reliable therapy recommendations and to decide on adequate measures for infection prevention and control. For conventional MRSA, rapid *mec* gene genotyping tools are available, which enable diagnosis typically 24–48 h before phenotypic OXA-resistance results. Currently, however, this is not possible for MRLM strains due to the high *gdp*P diversity. Clinical studies investigating the actual effectiveness of β-lactams (and alternative antibiotics) in controlling MRLMs are, therefore, essential.

## Supplementary Data

Supplementary material 1

Supplementary material 2
