# Supplementary material for: Mutations in the gdpP gene are a clinically relevant mechanism for β-lactam resistance in meticillin-resistant Staphylococcus aureus lacking mec determinants
Source: Microb Genom. 2021 Sep 6;7(9):000623. doi: 10.1099/mgen.0.000623 (PMC8715439; doi:10.1099/mgen.0.000623)
Supplement: Supplementary material 1 [file mgen-7-0623-s001.pdf]

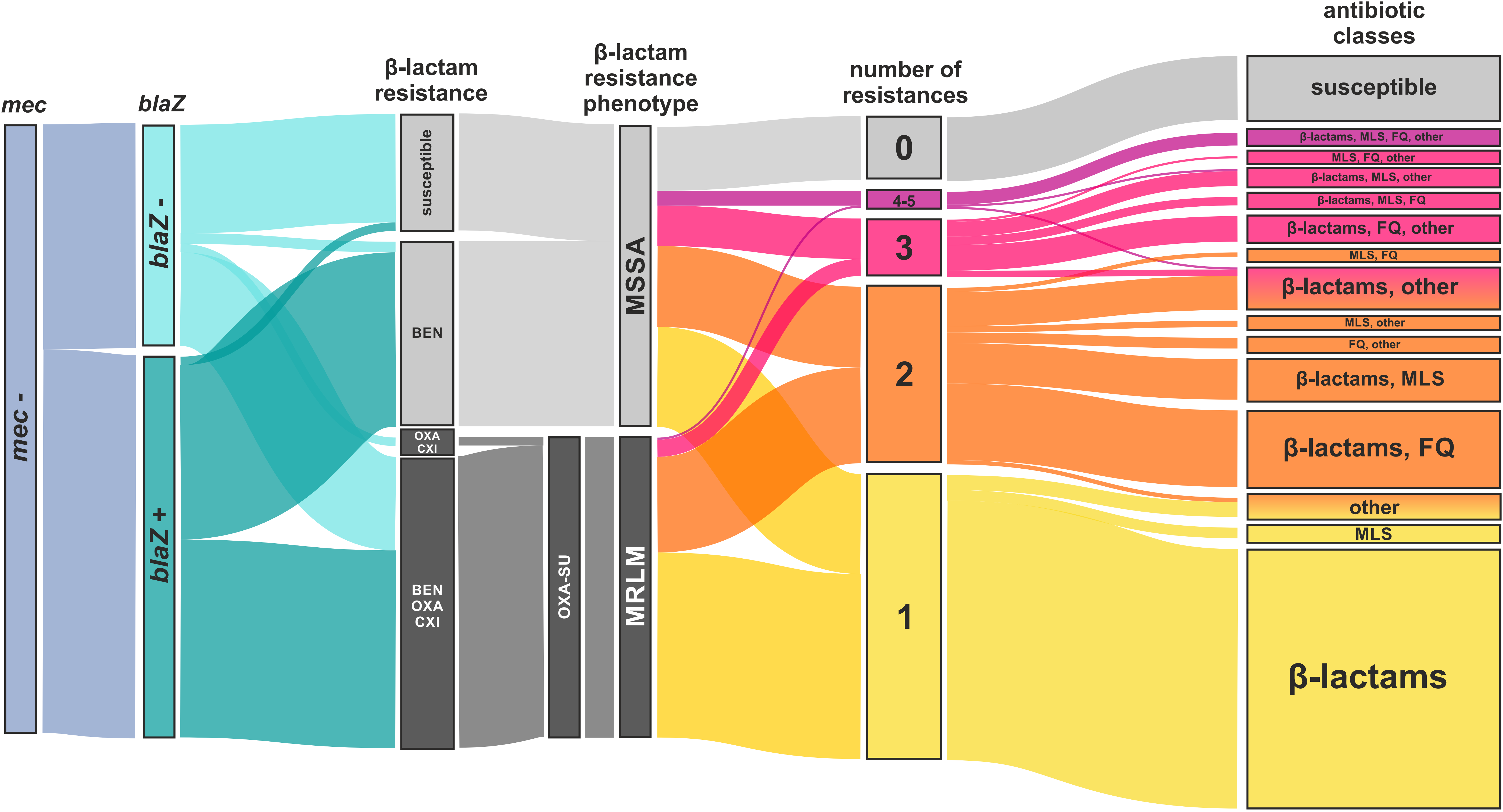

Supplementary file 2. Resistance characteristics of 283 *S. aureus* isolates; presence of *mec* genes and *blaZ* as determined by PCR and extracted from genome data, respectively; phenotypic resistance towards β-lactam antibiotics; number and type of antibiotic classes affected by resistance. FQ, fluoroquinolones; MLS, macrolides, lincosamides and streptogramins. Flow between categories is indicated by connecting lines. Line width is proportional to quantity.
